# Total Intravenous Anesthesia Using Target-Controlled Infusion with Propofol for Category 1 Emergency Cesarean Section in Patients with Preeclampsia with Severe Features

**DOI:** 10.3390/life15081237

**Published:** 2025-08-04

**Authors:** Janos Szederjesi, Emoke Almasy, Oana Elena Branea, Matild Keresztes

**Affiliations:** 1 Department of Anesthesiology and Intensive Care, George Emil Palade University of Medicine, Pharmacy, Science and Technology of Târgu Mureș, 540139 Târgu Mureș, Romania; yangzi37@gmail.com (J.S.); almasyemoke@yahoo.com (E.A.); branea_oana@yahoo.com (O.E.B.); 2 Department of Anesthesiology and Intensive Care, County Emergency Clinical Hospital of Târgu Mureș, 540136 Târgu Mureș, Romania

**Keywords:** preeclampsia, c-section, propofol, TIVA-TCI, Marsh model

## Abstract

Preeclampsia with severe features presents major anesthetic challenges, particularly in category 1 cesarean sections, in which rapid, safe, and hemodynamically stable induction is critical. Neuraxial techniques may be controversial due to neurological symptoms, making general anesthesia a viable option. However, traditional general anesthesia may exacerbate hypertension and increase maternal and fetal risks. Two primigravida patients with elevated blood pressure and neurological symptoms underwent category 1 cesarean delivery under TIVA-TCI with propofol, using the Marsh model. Hemodynamic stability, drug dosing, and maternal–neonatal outcomes were monitored. Sufentanil was administered for analgesia; neuromuscular blockade was achieved with rocuronium and reversed with sugammadex. No BIS or TOF monitoring was available. Both patients maintained stable hemodynamics and oxygenation throughout surgery. Intubation was successfully performed at an effect-site concentration of 3.5 µg/mL. Neonatal Apgar scores were within acceptable limits. No major complications occurred intraoperatively or postoperatively. TCI allowed individualized dosing and smooth emergence. TIVA-TCI with propofol appears to be a viable alternative to volatile-based general anesthesia in category 1 emergencies for cesarean sections for patients with preeclampsia with severe features, especially when neuraxial anesthesia is controversial. It offers hemodynamic stability and controlled depth of anesthesia, though its use requires experience and may not be optimal in cases requiring ultra-rapid induction.

## 1. Introduction

Hypertensive emergencies during pregnancy are among the most critical obstetric complications, alongside hemorrhagic emergencies. Preeclampsia and eclampsia are the most concerning forms, as they significantly contribute to both maternal and fetal morbidity and mortality [1,2,3].

Early recognition and prompt management of hypertensive emergencies are essential for maternal and neonatal well-being. Successful outcomes rely on a coordinated, multidisciplinary approach involving anesthesiologists, cardiologists, obstetricians, neonatologists, and nurses. Raising awareness about identifying abnormal blood pressure and neurological signs indicative of severe disease is vital. Neurological symptoms are often the most readily detectable, whereas hepatic, hematologic, or renal dysfunction may be diagnosed later due to delays in obtaining laboratory and urine test results [2,3,4].

Hypertensive emergencies arise from hypertensive disorders of pregnancy, which are classified into four categories: chronic hypertension, gestational hypertension, preeclampsia–eclampsia, and preeclampsia superimposed on chronic or gestational hypertension [1,2].

The modern definition of preeclampsia, as outlined by the American College of Obstetricians and Gynecologists (ACOG, 2020) [5], is as follows [3,6]:

1. New-onset hypertension that occurs most often after 20 weeks of gestation and frequently near term. Blood pressure of ≥140 mm Hg systolic or ≥90 mm Hg diastolic on two occasions at least 4 h apart.
2. Proteinuria, which is usually present but not mandatory for diagnosis.

Instead of the previous classification of “severe preeclampsia,” the condition is now categorized as preeclampsia with or without severe features, with severe features indicating end-organ dysfunction [6,7].

Anesthesia in preeclampsia with severe features is highly challenging, especially in category 1 cesarean sections (c-sections), where time pressure and anesthesia choice are critical. While spinal anesthesia is preferred, its use becomes questionable in cases of neurological dysfunction, which may indicate raised intracranial pressure [8,9,10,11]. However, general anesthesia may provoke hypertensive surges and intracerebral complications [9,12,13].

Total intravenous anesthesia (TIVA) with target-controlled infusion (TCI) has gained increasing popularity over recent decades, driven by advancements in pharmacokinetic models and infusion technology. While guidelines such as the “Guidelines for the Safe Practice of Total Intravenous Anaesthesia (TIVA)” have been developed to standardize and optimize its use, evidence and formal recommendations for its routine application in obstetric anesthesia remain limited [14].

The level to which it impacts obstetrical medicine is uncertain, as the current literature lacks sufficient data to support this approach. TIVA-TCI offers significant advantages for both frail and obstetric patients by enabling the precise control of anesthetic depth and minimizing hemodynamic disturbances [15,16]. In frail patients, who often have reduced physiological reserves, TIVA-TCI allows for gentle induction and maintenance of anesthesia with a predictable and safe recovery profile [15]. For obstetric patients, TIVA-TCI helps avoid the uterine relaxation and cardiovascular effects associated with volatile anesthetics, contributing to safer maternal and neonatal outcomes [16]. Compared with manual dosing, which can lead to small variations with potentially pronounced effects, TCI technology provides precise and consistent drug delivery, making it particularly valuable in sensitive patient populations.

Studies evaluating TIVA-TCI in cesarean delivery are mainly observational, often focusing on propofol and remifentanil protocols, with limited data specifically addressing high-risk populations such as those with preeclampsia with severe features.

Our case reports add to the existing literature by describing the use of TIVA-TCI in two patients with preeclampsia with severe features undergoing emergency cesarean section. It provides detailed perioperative management, including adjustments made for full stomach status, induction techniques, and postoperative recovery. This report highlights practical considerations and contributes novel clinical observations to an area where prospective studies are lacking.

## 2. Case Series Presentation

We present two cases of preeclampsia with severe features from County Emergency Clinical Hospital of Targu Mures. The hospital’s ethics committee approved the presentation.

In our hospital, category 1 emergency cesarean sections are typically managed with balanced general anesthesia, using rapid sequence induction with propofol and rocuronium, combined with a low dose of opioid, and maintenance with sevoflurane as the primary volatile agent.

In these cases, the obstetric anesthetist opted for TIVA using propofol 1% administered via TCI pump (Braun Perfusor Space, Melsungen, Germany). The Marsh pharmacokinetic model was selected to reduce the time required for programming the infusion pump, and because local experience with this model is more extensive.

The anesthetic/surgical protocol used was as follows:

- The patient positioning in a left lateral tilt.
- Establishing two peripheral intravenous lines.
- Initiation of crystalloid administration with approximately 500 mL of Ringer’s solution.
- Oxygen administration via face mask, 5–6 L/min, as part of the preoxygenation process.
- Prokinetic administration: metoclopramid 10 mg intravenous (IV).
- Continuous monitoring included heart rate via ECG monitoring, oxygen saturation, and noninvasive blood pressure.
- Insertion of urinary catheter.
- Surgical preparation and draping were performed before induction.
- Sufentanyl (10 mcg) was administered before propofol initiation.
- Initial TCI target plasma concentration (Cpt) was set at 7–8 mcg/mL to rapidly increase the effect-site concentration (Ce).
- As the Ce increased to 3 mcg/mL, rocuronium was administered at a dose of 1–1.2 mg/kg.
- Orotracheal intubation at Ce 3.5 mcg/mL, using a C-MAC videolaryngoscope blade (Storz C-MAC 8403 ZX, Karl Storz SE & Co. KG, Tuttingen, Germany), and using a 7.0 mm endotracheal tube fixed at 20–21 cm. During intubation, bag-mask ventilation was omitted, and Sellick’s maneuver was not performed.
- After intubation, Cpt was reduced to 4 mcg/mL.
- After intubation, a nasogastric tube was placed, and gastric contents were aspirated.
- Pfannenstiel incision was made as soon as the cuff passed through the vocal cords and was inflated.
- Anesthesia was maintained at a Ce of 4 mcg/mL.
- Sufentanil’s second dose (10 mcg) was administered after delivery, and third dose as needed.
- Target-controlled infusion of propofol was stopped (Cpt set to 0 mcg/mL) upon completion of wound closure.
- Plasma concentration and effect-site concentration were monitored continuously until extubation.
- Neuromuscular blockade was reversed with sugammadex 100–200 mg IV.
- Extubation: During emergence, external stimulation was avoided, and extubation was performed following spontaneous eye opening, consistent with awake extubation practices as recommended by the DAS Extubation Guidelines [17].
- For analgesia, the patient received intravenous paracetamol 1 g, ibuprofen 300 mg, metamizole 1 g, and tramadol 100 mg, along with granisetron 1 mg administered as an antiemetic, on extubation.
- No Train-of-Four (TOF) monitoring or depth of anesthesia assessment using Bispectral Index (BIS) or Entropy was performed during the intervention, primarily due to the lack of routine availability of these monitoring modalities.

Case 1: A 21-year-old primigravida, at an estimated gestational age of 37–38 weeks based on ultrasound evaluation, presented to the obstetric emergency department in January 2025, following the spontaneous rupture of membranes accompanied by elevated blood pressure. The pregnancy was unmonitored, as the patient had not undergone prenatal care or previous obstetric assessments. The initial blood pressure recorded by the emergency medical team was 206/128 mmHg.

The patient was admitted to the obstetrics department with a diagnosis of premature rupture of membranes and suspected gestational hypertension. She had no significant medical history, no previous surgeries, and no known drug or food allergies. Her weight was 90 kg, and her height was 165 cm, and BMI was 33.1 kg/m^2^.

On admission, cervical secretions were cultured. Complete blood count, biochemistry, coagulation panel, and urinalysis were unremarkable, with normal hemoglobin, platelets, and liver function and no proteinuria.

Cardiology consultation recommended the initiation of methyldopa 250 mg per os (p.o.) every six hours, along with nifedipine 20 mg p.o. as needed for blood pressure readings exceeding 140/90 mmHg.

After 24 h, the patient went into labor and developed hypertension exceeding 180/93 mmHg during labor, which was unresponsive to nifedipine.

An obstetric ultrasound examination was performed, revealing no signs of retroplacental hematoma, uteroplacental apoplexy, or premature placental detachment.

The anesthetist evaluated the patient, noting a severe headache (onset within 40 min), visual disturbances, epigastric and right upper quadrant pain, and a blood pressure of 180/100 mmHg. Magnesium sulfate (MgSO_4_) 4 g was administered in two divided doses, and a category 1 c-section was decided.

Preoperative fasting status: the patient had consumed solid food 6 h before surgery and clear liquids less than 2 h before surgery.

Table 1 outlines chronologically the events from the patient’s entry into the operating room until awakening, detailing the timing and administration of muscle relaxants and the initiation of TIVA with propofol.

A male newborn was delivered with a birth weight of 2780 g, an Apgar score of 7/1 and 9/5, and the presence of nuchal cord (umbilical cord wrapped around the neck) at the moment of delivery. Postoperative examination of the placenta revealed no abnormalities.

Following extubation, the patient was transferred to the obstetric intensive care unit with stable spontaneous respiration and preserved neurological function. Postoperative pain was assessed using the Numeric Rating Scale (NRS). Upon admission, the patient’s NRS pain score was 5, indicating moderate pain intensity. On admission, her blood pressure was 162/108 mmHg, heart rate 76 bpm, and oxygen saturation 95% with room air.

On Day 0, the patient’s blood pressure was managed with methyldopa 250 mg p.o. every eight hours and a 24 h MgSO_4_ infusion (1 g/h). She was neurologically stable, with no headaches or visual disturbances, and was mobilizing independently. She was transferred to the ward on postoperative Day 1, where blood pressure remained controlled and methyldopa was continued.

The patient was discharged on postoperative day 5. Total length of stay in hospital: 7 days.

Case 2: A 24-year-old primipara was admitted to the obstetrics clinic for management of a post-term pregnancy at 40–41 weeks of gestation. Upon presentation in February 2025, the fetus was in cephalic presentation, membranes were intact, and labor had not yet commenced.

The patient weighed 95 kg, was 168 cm tall, had a BMI of 33,7 kg/m^2^, had no known allergies, and had been on chronic treatment with iron (Sideral) tablets. Upon admission, her blood pressure was 220/130 mmHg, and her heart rate was 105 bpm, raising suspicion of gestational hypertension.

A complete blood count (CBC), coagulation panel, and biochemistry tests were collected, revealing no pathological abnormalities, except for a mild leukocytosis (11,800/µL) and a hemoglobin level of 11.20 g/dL.

An obstetric ultrasound examination was performed, revealing no signs of retroplacental hematoma, uteroplacental apoplexy, or premature placental detachment.

During the anesthetic evaluation, the patient reported experiencing visual disturbances, specifically blurred vision, for the preceding 24 h, without associated headaches. Physical examination revealed lower limb edema, and her blood pressure remained elevated at 185/115 mmHg. Then, 2 g of IV MgSO_4_ was administered.

The patient had consumed food 2.5 h prior and liquids 1 h before the consultation.

To mitigate the risk of pulmonary aspiration in both patients, a series of precautionary measures was implemented. Induction with TIVA-TCI using propofol was initiated only after the obstetric team had completed patient isolation and confirmed surgical readiness. No external stimulation was applied during this phase to avoid triggering airway reflexes. Positive pressure ventilation was deliberately avoided to minimize the risk of gastric insufflation. Spontaneous ventilation was preserved throughout the initial phase of induction. Once the propofol effect-site concentration reached 3.0 µg/mL, rocuronium was administered to achieve neuromuscular blockade and allow for secure airway management. This approach facilitated a smooth and controlled induction while minimizing the aspiration risk, particularly in the context of an unfasted obstetric patient.

Table 2 outlines chronologically the events from the patient’s entry into the oper-ating room until awakening, detailing the timing and administration of muscle relaxants and the initiation of TIVA with propofol.

A male newborn was delivered, weighing 3790 g and measuring 57 cm in length, with an Apgar score of 9/1 and 9/5. Postoperative examination of the placenta revealed no abnormalities.

Although the average blood loss following lower-segment cesarean section (LSCS) is approximately 1000 mL, it can vary based on multiple factors, including the operator’s experience. In our cases, the first procedure was performed by a young practitioner, whereas the second was conducted by an experienced practitioner, which may have contributed to the differences observed in blood loss.

Upon transfer, her blood pressure was 138/99 mmHg, her heart rate was 70 bpm, and she had spontaneous, effective respirations. Her SpO_2_ was 95% with nasal cannula oxygen at 2L/min. Postoperative hypertension treatment included methyldopa 250 mg p.o. every six hours, nifedipine 20 mg p.o. every twelve hours, and MgSO_4_ IV 1 g/h for 24 h postoperatively.

Two hours postoperatively, the patient developed uterine hypotonia without associated hemodynamic instability. Following obstetric reassessment, carboprost 100 mcg IV was administered along with an additional 10 IU of oxytocin via continuous intravenous infusion.

By postoperative day two, her visual disturbances had completely resolved, and blood pressure was effectively controlled with antihypertensive therapy consisting of methyldopa and nifedipine. However, her hemoglobin level decreased significantly to 6.79 g/dL, necessitating the transfusion of one unit (350 mL) of 0-positive packed red blood cells, administered according to the patient’s blood type compatibility. Following the transfusion, the patient was transferred to the obstetrics ward for continued monitoring and further recovery.

On postoperative day 7, the patient requested discharge against medical advice despite the current physician’s recommendations. Total length of stay in hospital: 7 days.

## 3. Discussion

The guideline titled “Total Intravenous Anaesthetic (TIVA) for the Obstetric Population” (document code: WAHT-TP-094) was developed and approved by the Worcestershire Acute Hospitals NHS Trust in the UK [16]. It discusses the application of TIVA in obstetric patients, highlighting its benefits such as a minimal impact on uterine tone, reduced bleeding risk, and lower incidence of postoperative nausea and vomiting. These advantages are particularly relevant for patients with preeclampsia, where maintaining hemodynamic stability is crucial. Regarding the pharmacological model, the guideline does not mandate a single approach but provides detailed guidance. Model selection should be based on clinical experience, patient-specific factors, and the available equipment; in the presented cases, the choice of the Marsh model reflects local clinical familiarity and practice [16].

TCI-based induction, as supported by this guideline, involves initiating a high infusion rate via the TCI pump (e.g., 1200 mL/h), followed by a rapid reduction to the maintenance rate. This method targets plasma concentrations between 4 and 6 mcg/mL, typically using the Marsh model, and is considered more suitable for clinicians with greater experience in obstetric TIVA. Given the local clinical familiarity with TIVA-TCI, this approach was chosen over rapid induction with a propofol bolus followed by TCI maintenance. However, induction times were prolonged (2 min 45 s and 3 min 12 s, respectively). Thus, the described technique is not recommended in clinical scenarios requiring rapid induction, such as fetal bradycardia or active hemorrhage.

The pharmacological Marsh model applied in these cases aligns with data from the OBSTIVA-UK prospective study, in which the Marsh model was utilized more frequently compared to the Schnider model (64 vs. 36 cases). Additionally, the propofol maintenance dose corresponded to the plasma concentrations employed in the presented cases. In contrast to the OBSTIVA-UK study, manual boluses were not administered; instead, an increased target plasma concentration was selected to facilitate a more rapid increase in effect-site concentration [18].

In the ObsTIVA-UK study, remifentanil was utilized in 84% of obstetric TIVA cases. Regarding neonatal outcomes, 73% of neonates had an Apgar score below 7 at 1 min, and 17% at 5 min. No neonates scored below 7 at 10 min. This suggests a relatively high incidence of initial neonatal compromise, emphasizing the need for careful consideration when using remifentanil during obstetric TIVA [18].

A systematic review by Arielle Maroni et al. concluded that remifentanil is effective and generally safe in preterm and term neonates during the immediate postoperative period. However, potential complications such as chest wall rigidity were noted, and the authors emphasized that long-term neonatal safety data remain scarce [19].

In the presented cases, remifentanil was not used due to hospital availability constraints. Instead, sufentanil 10 mcg was administered before induction and again after delivery. In the first neonate, the Apgar scores were 7 at 1 min and 9 at 5 min, possibly related to an umbilical cord wrapped around the neck. In the second neonate, Apgar scores were 9 at 1 min and 9 at 5 min.

Krishna and colleagues observed that sufentanil administration during cesarean delivery under general anesthesia provided effective maternal analgesia without significant neonatal depression. Although minor respiratory depression was noted briefly in neonates, Apgar scores remained clinically acceptable, indicating sufentanil’s relative safety when carefully administered during cesarean sections [20,21].

Propofol, by its lipophilic nature, facilitates rapid placental transfer, leading to detectable concentrations in the fetal circulation. Studies have reported umbilical venous concentrations approximately 22–32% of maternal levels at delivery. Despite this transfer, research indicates that propofol’s effects on neonates are minimal and transient. Newborns typically exhibit high Apgar scores, suggesting effective post-delivery adaptation. However, factors such as maternal dosing, timing between drug administration and delivery, and individual patient characteristics can influence fetal exposure. Therefore, careful dosing and timing are essential to minimize potential neonatal impacts [22].

In comparing propofol dosing between the manual bolus technique and TIVA-TCI, several studies have observed differences in total propofol consumption and recovery times. For instance, in the study of Ferreira Laso and colleagues comparing TCI and manual infusion techniques, they found that the total propofol dose was lower in the TCI group (112.4 ± 60.9 mL) compared to the manual group (133.8 ± 80.3 mL), though the difference was not statistically significant (*p* = 0.241). They also noted that the recovery time was significantly shorter in the TCI group (7.48 ± 3.1 min) compared to the manual group (10.3 ± 4.9 min) (*p* = 0.008) [23].

Serap Aktas Yildirim and colleagues conducted a study comparing TCI with the manual bolus administration of propofol. Their findings demonstrated that TCI provides improved cardiovascular stability, facilitating smoother induction and faster recovery. Although the study does not specifically address preeclamptic patients, the results are highly relevant due to the hemodynamic sensitivity and risks associated with preeclampsia [24].

In the presented cases, induction was performed with propofol using a target effect-site concentration of 3.5 mcg/mL with the TCI technique. The calculated propofol doses at the time of rocuronium administration were 2.75 mg/kg in the first case and 2.55 mg/kg in the second case, both of which exceeded the typical induction range of 1.5–2 mg/kg associated with manual bolus administration.

Based on the recommendation by Tushar M. Chokshi, intubation can be effectively performed at a TCI propofol target concentration of 3.5–4 mcg/mL. In the presented cases, intubation was initiated at a concentration of 3.5 mcg/mL, consistent with this guidance [25].

Considering the inherent risk of pulmonary aspiration in obstetric patients undergoing general anesthesia, particular attention was directed toward optimizing airway management and anesthetic technique. The first patient met standard fasting recommendations—6 h for solids and 2 h for clear fluids. In contrast, the second patient was classified as having a full stomach, having fasted for only 2.5 h for solids and 1 h for clear liquids, thereby presenting a significantly increased risk of aspiration. Propofol offers several advantages as a hypnotic agent, particularly in the context of airway management and pharmacological profile. It is non-irritating to the airway, does not provoke coughing, reduces the incidence of laryngospasm, and possesses intrinsic antiemetic and anxiolytic properties, thereby contributing to a smoother induction. However, at effect-site concentrations of 3.0 µg/mL or higher, propofol reliably induces apnea, requiring timely airway protection. As described by Al-Rifai and Mulvey, spontaneous ventilation is generally preserved up to a concentration of 3.0 µg/mL, with apnea becoming increasingly likely at or above this level [26].

A well-recognized concern with inhalational anesthesia during obstetric surgery is dose-dependent uterine relaxation, which can lead to an increased risk of postpartum hemorrhage [27]. In contrast, TIVA offers the benefit of maintaining uterine tone, as agents like propofol do not have uterine-relaxant effects [28].

A key limitation in the management of these cases was the absence of BIS and TOF monitoring, which would have provided the objective assessments of anesthetic depth and neuromuscular blockade. Both cases were classified as category 1 cesarean sections due to the presence of severe, uncontrolled hypertension, representing an immediate threat to maternal health. However, there were no additional features of extreme urgency, such as fetal bradycardia or significant maternal hemorrhage. Despite the high-risk classification, the time to delivery was relatively prolonged, i.e., 19 and 11 min, respectively, which may be considered suboptimal in more acute obstetric emergencies. These limitations should be taken into account when assessing the broader applicability of TIVA-TCI techniques in time-sensitive scenarios. Nevertheless, both cases had favorable maternal outcomes, including the complete remission of neurological symptoms. In clinical situations where tight blood pressure control is critical, the TIVA-TCI approach using propofol proved to be an effective method for achieving gradual and stable hemodynamic management.

## 4. Conclusions

In summary, while direct studies on TIVA-TCI propofol use in severe preeclampsia are scarce, the existing literature suggests that TIVA, particularly with TCI, can offer hemodynamic stability and other benefits in the obstetric population. However, in other obstetric emergencies—such as cases involving fetal bradycardia or active hemorrhage—manual induction with a rapid bolus of propofol may be more appropriate, as it can further reduce the decision-to-delivery interval. Intermittent sufentanil administration during cesarean section may offer effective intraoperative analgesia with minimal neonatal depression, particularly when repeated dosing is carefully timed after delivery.

## Figures and Tables

**Table 1:** Chronological events from entry into the operating room until awakening.

	Entering OR + Monitor, Draping	TCI Start	Rocuronium Adm.	OTI + Incision	Delivery	Stop Propofol Infusion	Extubation	Total
Time	12:40	12:47	12:49	12:50	12:59	13:33	13:53	
Cpt (μg/mL)	0	8	8	8	4	4	1.7	
Ce (μg/mL)	0	0	3	3.5	4	4	1.93	
Propofol (mg)		0	248	277 (from start)		696	0	973
Rocuronim (mg)			100					100
Sufentanil (µg)		10			10			20
BP (mmHg)	182/99	180/95	170/104	160/97	145/83	140/80	138/75	
MAP (mmHg)	126	123	126	118	105	102	96	
SpO_2_ (%)	99	99	98	99	99	99	98	
HR (/min)	90	93	100	115	98	90	75	
Urine output (mL)								300
Fluids (mL)								600
E.Blood loss (mL)								900
Oxytocin (U.I.)					5 + 10 continuous infusion			15 *

OR = operating room; Ce = effect-site concentration; Cpt = target plasma concentration; TCI = target control infusion; OTI = orotracheal intubation; BP = blood pressure; MAP = mean arterial pressure; HR = heart rate; E.Blood loss = estimated blood loss; SpO_2_ = saturation. * Infusion of oxytocin was not completed at extubation.

**Table 2:** Chronological events from entry into the operating room until awakening.

	Entering OR + Monitor, Draping	TCI Start	Rocuronium Adm.	OTI + Incision	Delivery	Stop Propofol Infusion	Extubation	Total
Time	11:35	11:40	11:42	11:43	11:46	12:22	12:38	
Cpt (μg/mL)	0	7	7	7	4	4	1.3	
Ce (μg/mL)	0	0	3	3.5	4	4	1.5	
Propofol (mg)		0	243	266 (from start)		773	0	1039
Rocuronium (mg)			100					100
Sufentanil (mcg)		10			10 + 10			30
BP (mmHg)	210/130	197/119	180/105	169/118	141/82	120/65	140/90	
MAP (mmHg)	157	145	130	135	102	83	107	
SpO_2_ (%)	99	98	98	99	98	99	99	
HR (/min)	120	117	120	128	85	90	80	
Urine output (mL)								200
Fluids (mL)								1000
E.Blood loss (mL)								600
Oxytocin (U.I)					5 + 10 continuous infusion			15 *

OR = operating room; Ce = effect-site concentration; Cpt = target plasma concentration; TCI = target control infusion; OTI = orotracheal intubation; BP = blood pressure; MAP = mean arterial pressure; HR = heart rate; E.Blood loss = estimated blood loss; SpO_2_ = saturation. * Infusion of oxytocin was not completed at extubation.

## Data Availability

The data used for this study can be found in the database of the Târgu Mureș, County Emergency Clinical Hospital, Romania.
